# Applying the quality improvement collaborative method to process redesign: a multiple case study

**DOI:** 10.1186/1748-5908-5-19

**Published:** 2010-02-25

**Authors:** Leti Vos, Michel LA Dückers, Cordula Wagner, Godefridus G van Merode

**Affiliations:** 1 NIVEL, Netherlands Institute for Health Services Research, P.O. Box 1568, 3500 BN Utrecht, the Netherlands; 2 Impact, Dutch knowledge and advice center for post-disaster psychosocial care, P.O. Box 78, 1110 AB Diemen, the Netherlands; 3 Institute for Health and Care Research, Department of Public and Occupational Health, VU University Medical Centre Amsterdam, P.O. Box 7057, 1007 MB Amsterdam, the Netherlands; 4 Care and Public Health Research Institute (CAPHRI), Maastricht University Medical Centre+, P.O. Box 5800, 6202 AZ Maastricht, the Netherlands

## Abstract

**Background:**

Despite the widespread use of quality improvement collaboratives (QICs), evidence underlying this method is limited. A QIC is a method for testing and implementing evidence-based changes quickly across organisations. To extend the knowledge about conditions under which QICs can be used, we explored in this study the applicability of the QIC method for process redesign.

**Methods:**

We evaluated a Dutch process redesign collaborative of seventeen project teams using a multiple case study design. The goals of this collaborative were to reduce the time between the first visit to the outpatient's clinic and the start of treatment and to reduce the in-hospital length of stay by 30% for involved patient groups. Data were gathered using qualitative methods, such as document analysis, questionnaires, semi-structured interviews and participation in collaborative meetings.

**Results:**

Application of the QIC method to process redesign proved to be difficult. First, project teams did not use the provided standard change ideas, because of their need for customised solutions that fitted with context-specific causes of waiting times and delays. Second, project teams were not capable of testing change ideas within short time frames due to: the need for tailoring changes ideas and the complexity of aligning interests of involved departments; small volumes of involved patient groups; and inadequate information and communication technology (ICT) support. Third, project teams did not experience peer stimulus because they saw few similarities between their projects, rarely shared experiences, and did not demonstrate competitive behaviour. Besides, a number of project teams reported that organisational and external change agent support was limited.

**Conclusions:**

This study showed that the perceived need for tailoring standard change ideas to local contexts and the complexity of aligning interests of involved departments hampered the use of the QIC method for process redesign. We cannot determine whether the QIC method would have been appropriate for process redesign. Peer stimulus was non-optimal as a result of the selection process for participation of project teams by the external change agent. In conclusion, project teams felt that necessary preconditions for successful use of the QIC method were lacking.

## Background

**Figure 1:**
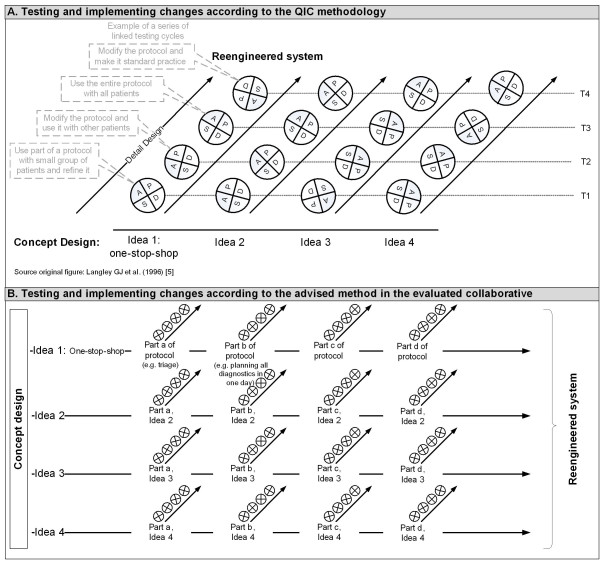
Testing and implementing changes using PDSA cycles

**Figure 2:**
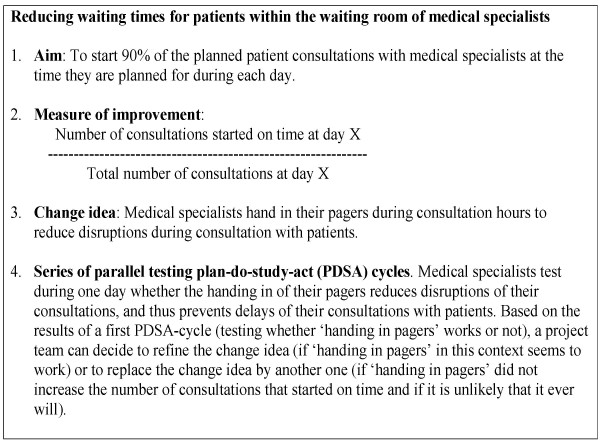
Applying the model for improvement, an example

Quality improvement collaboratives (QICs) are used increasingly in many countries to achieve large-scale improvements in performance and to provide specific remedies to overcome the typically slow diffusion of medical and healthcare innovations [1-3]. A QIC is a multifaceted method that seeks to implement evidence-based practice through sharing knowledge with others in a similar setting over a short period of time [4]. Within the QIC method, external change agents provide collaborative project teams from different healthcare departments or organisations with a clear vision for ideal care in the topic area and a set of specific changes that may improve system performance significantly [5,6]. Project teams also learn from the external change agent about the model for improvement. The model for improvement incorporates four key elements [6]: specific and measurable aims; measures of improvement that are tracked over time; key changes that will result in the desired improvement; and series of parallel testing plan-do-study-act (PDSA) cycles. Each series involves a test of one change idea (Figure 1, part A) [7]. On the basis of the results of the first test of one series, a project team can decide to refine the change idea (in case the change idea works in their context) or to start a new test series of a new change idea (in case the test did not lead to the desired result). These PDSA cycles should be short but significant, testing a big change idea in a short timeframe so that a team can identify ways to improve or change the idea [8]. In Figure 2, an example is given to illustrate the model for improvement.

In addition to the relatively efficient use of external change agent support and the exchange of change ideas as well as the model for improvement, the strength of the QIC method seems to be that collaborative project teams share experiences of making changes, which accelerates the rate of improvement (peer stimulus) [3].

However, despite the widespread use of QICs, a recent review on their impact indicates that evidence is positive but limited, and the effects cannot be predicted with certainty [5]. This apparent inconsistency requires a deeper understanding of how and why QICs work. Therefore it is necessary to explore the 'black box' of the intervention and to study the determinants of success or failure of the QIC method [5,9]. In this article, we contribute to this by assessing the applicability of this quality improvement method to process redesign. Process redesign aims to improve the organisation of care delivery in terms of waiting times in a patients' care trajectory. From other studies it is already known that the QIC method can be successfully applied to improve the organisation of care delivery in specific departments, such as emergency and surgery departments [8,10]. But, to our knowledge, it is unknown whether the QIC method itself is applicable for implementing complex process redesigns, which aim to change patterns of interaction between departments in order to achieve speedy and effective care from a patient's perspective [11]. Therefore, we explored in this study whether the QIC method was applied to complex process redesign projects in a process redesign collaborative in the Netherlands.

## Methods

The collaborative described in this paper was part of the Dutch national quality improvement programme 'Sneller Beter' ('Better Faster'), which began in 2004 as an initiative from the Ministry of Health and the Dutch Hospital Association. 'Sneller Better' aimed to realise substantial and appealing performance improvements in three groups of eight Dutch hospitals in the areas of patient logistics and safety. These twenty-four hospitals were enrolled in the programme by a selection procedure that assessed the organisational support, commitment for participation, availability of personnel, time to realise improvements, and experience with improvement projects. Each group of eight hospitals joined the programme for two years (2004 to 2006, 2005 to 2007, or 2006 to 2008) and participated in several QICs on different topics (*e.g.*, pressure ulcers, process redesign) [12].

The process redesign collaborative evaluated in this study represented the third group of eight hospitals. The overall aim of this collaborative was to reduce the time between the first visit to the outpatients clinic and the start of treatment and/or to reduce the length of in-hospital stay by 30% for selected patient groups [13]. Eighteen project teams from the eight participating hospitals joined this collaborative, which started in October 2006. Seventeen of these teams agreed to participate in our independent evaluation. The enrolment of project teams within the evaluated QIC differed per hospital. Project teams took part on their own initiative or were enrolled by the hospital board, but always in agreement with the external change agent.

### Process redesign collaborative

**Figure 3:**
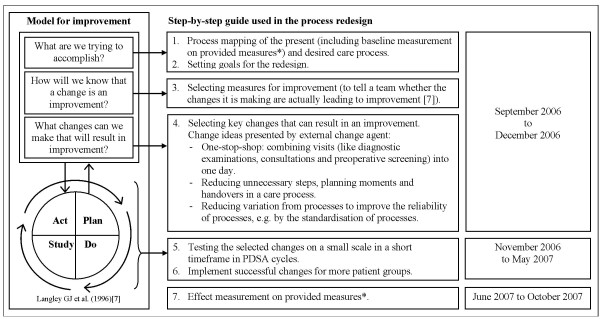
Step-by-step guide used in the process redesign collaborative including the model for improvement * The provided outcome measures were: 1) access time to outpatients clinic, 2) duration of diagnostic trajectory, 3) time between diagnosis and treatment, and 4) length of in-hospital stay. The provided intermediate measure (an indicator of progress [21]) was the number of visits to the outpatients clinic up to the start of treatment.

The evaluated collaborative used a step-by-step guide, which included the model for improvement (see Figure 3). This step-by-step guide was provided by the external change agent. Next to this, the external change agent organised five collaborative meetings to inform teams about the step-by-step guide as well as about changes that have worked at other sites. The presented evidence for improvement focused mainly at the introduction of a one-stop-shop, in which various visits per patient (diagnostic examinations, consultations, and preoperative screening) are planned for a single day, with the aim of reducing the throughput time of the diagnostic trajectory. Examples of other process redesign change ideas that were provided are: the standardisation of care processes in order to reduce variation, the reduction of the number of unnecessary steps in care processes (do not provide care for which there is no evidence of efficacy), the reduction of the number of planning moments or handovers in a care process so that fewer health care workers are involved in the process, and that each worker is involved only once per iteration of a process.

The change agent also provided a website enabling project teams to share information. Although it is recommended for QICs to test a big change idea in one series of testing cycles [8], the external change agent advised splitting up every planned change into smaller ones that could be tested instantaneously in a series of testing cycles based on their experiences of other collaboratives ( Figure 1, part B). By doing so, the external change agent tried to ensure that teams spent their initial resources on testing changes instead of dealing with barriers and resistance to change.

### Data collection

To explore the applicability of the QIC method, we evaluated the process redesign collaborative in a multiple case study design [14] using complementary qualitative data collection methods.

We analysed the process redesign team education manual to learn more about the provided change ideas and step-by-step guide. Further, we held a survey among hospital staff members who took part in the project implementations (project staff members) (n = 17) and among project leaders (n = 17) to gather data on project characteristics and aims, composition of the project teams, and project plans (including (planned) changes, project progress, and the application of the model for improvement). The surveys also included questions about team organisation (including a clear task division, self responsibility for progress, good compliance to arrangements, good communication and coordination, be in charge of implementation), organisational support (including support of strategic management, organisational willingness to change) and external change agent support (including sufficient support and supply of instruments, transfer of valuable insights), because it is known from literature that these are preconditions for successful use of the QIC method [12,15,16]. In the survey among project leaders, we included a validated questionnaire to assess these three preconditions [15]. Project staff members were asked to rate the amount of organisational support and external change agent support on a scale of 0 to 10. Questionnaires were sent to respondents one year after the start of the collaborative (September 2007), and sixteen project staff members (response = 94%) and eleven project leaders (response = 65%) completed and returned them.

We also interviewed all project staff members (n = 17) after they returned the questionnaire between October and December 2007. Interview themes were: change agent support (provided best practices, change concepts, and quality improvement methods), shared experiences between teams, and applicability of the model for improvement.

In addition, we observed the guidance and training offered by the external change agent during meetings and training sessions of the process redesign collaborative. The observations provided us context for the analysis of the questionnaires and interviews.

Finally, we analysed the results reported on the outcome and intermediate measures set by the external change agent, who collected these results in a 'Sneller Beter' database and, at our request, provided us with these data (December 2007).

All gathered information was used to describe the collaborative process and to assess the applicability of the QIC method to process redesign. Additional information about the preconditions was gathered to evaluate whether they could have influenced the results.

## Results

### Characteristics of the process redesign projects within the collaborative

Table 1 gives an overview of the characteristics of the process redesign projects. Fifteen project teams chose to redesign an elective care process. Eight of those projects involved care for cancer patients. Two project teams chose to redesign an acute care process.

**Table 1:** Characteristics of enrolled process redesign projects

		**Volume of patient group**^**1 **^**(patients/yr)**	**Acute (A) or elective (E)**^**1,2**^	**Process to be redesigned**^**1,2**^			Involved medical departments (description*, N**)^**1,2,3**^	
No.	Patient Group			Access to care	Diagnostic trajectory (outpatients clinic)	In-hospital stay		
1.	Acute stomach complaints	200	A	-	-	+	**Internal medicine**; **Radiology**; Pathology	3 (2)
2.	Breast cancer	120	E	-	+	-	**Oncology**; **Surgery**; Radiology	3 (2)
3.	Breast cancer	250	E	+	+	-	Oncology; Surgery; Radiology	3 (?)
4.	Chronic Obstructive Pulmonary Disease	?	E	-	+	+	**Lung diseases**	1 (1)
5.	Colon cancer	110	E	+	+	+	**Gastroenterology**; **Surgery**; **Oncology**; **Anaesthesiology**; Radiology	5 (4)
6.	Colon cancer	80	E	+	+	-	Gastroenterology; Surgery; Radiology; Pathology	4 (?)
7.	Colon cancer	150	E	-	+	-	**Gastroenterology**; **Surgery**; Radiology; Anaesthesiology; Oncology	5 (2)
8.	Head- and neck cancer	650	E	+	+	+	**Ear, Nose and Throat; Radiology**; **Jaw surgery**; **Radiotherapy**; Oncology; Pathology; **Anaesthesiology**; Plastic Surgery	8 (5)
9.	Hematuria	130	E	+	+	+	**Urology**; **Radiology**	2 (2)
10.	Lung cancer	400	E	-	+	-	**Lung diseases**; Radiology; Surgery; Pathology; Anaesthesiology	5 (1)
11.	Oesophageal atresia (children)	17	A	-	-	+	**Paediatric Surgery**; **Intensivis**t; Radiology	3 (2)
12.	Open Chest Surgery	1000	E	+	-	+	**Thorax Surgery**; **Anaesthesiology**	2 (2)
13.	Small Orthopaedic interventions	250	E	+	+	-	**Orthopaedics**; **Radiology**	2 (2)
14.	Small Orthopaedic interventions	>200	E	+	+	-	**Orthopaedics**; Anaesthesiology	2 (1)
15.	Benign Prostate Hypertrophy	100	E	-	+	+	**Urology**	1 (1)
16.	Colon cancer	100	E	+	+	+	**Surgery**; Gastroenterology; Radiology; Oncology	4 (1)
17.	Varicose veins	150	E	+	+	-	**Surgery**; **Dermatology**	2 (2)

+ Yes, - No; * in bold: medical departments that are represented by a medical specialist in the project team; ** number of medical departments involved (number of medical departments represented in project team). ^1^Data source: interviews among project staff members. ^2^Data source: survey among project staff members. ^3^Data source: survey among project leaders.

All project teams intended to make improvements in waiting times and delays, but in different areas (access times, throughput times of diagnostic trajectories, and/or length of stay) and for different types of patient groups. The median value of the volume of the involved patient groups was 150 patients a year (range 17 to 1,000). The number of medical departments involved in the redesigned care process was on average three and varied per project from one to eight departments. In seven instances, not all medical departments involved participated in the project team.

### Presence of preconditions for successful use of the QIC method

The project leaders and project staff members of six project teams shared the opinion that preconditions for successful use of the QIC method--*i.e.*, 'team organisation', 'organisational support', and 'external change agent support'--were sufficiently present (project no. 1, 4, 6, 10, 16, and 17). The remaining project teams show a diverse picture of the presence of the preconditions. In general, almost all project teams were positive about the organisation of their project team. One-half of the project teams had the opinion that support from their organisation and/or external change agent support was lacking.

### Evaluation of the collaborative process

This section describes the collaborative process according to the step-by-step guide provided to the process redesign collaborative (see figure 3).

### Step one

All projects started with a process analysis of the existing care process. Sixteen of the seventeen projects performed a baseline measurement.

### Step two

The baseline measurement and ideas about the desired care process formed the input for the project aims and changes that needed to be implemented. Although all project teams formulated project aims, only fourteen formulated at least one specific and measurable aim (range 0 to 7, average 2) (see Table 2).

**Table 2:** Application of the model for improvement in the enrolled process redesign projects

	Key elements of the model for improvement	**Specific and measurable aims (N)**^**1**^	**Measures of improvement**^**1**^				**Key changes**^**1,2,3**^			**PDSA**^**1,3**^	**Effect measurement (collaborative goals reached?)?**^**4**^
			Provided by external change agent		Established by the project team		Evidence for improvement (one-stop-shop) implemented in redesign?		Supplied change concepts used?		
No.	Patient Group		Outcome	Inter-mediate	Outcome	Process and/or intermediate	Yes/No	Comments			
1.	Acute stomach complaints	+ (1)	+	n.a.	+	+	-	n.a.	+	-	- (?)
2.	Breast cancer	- (0)	+	+	-	-	-	Already implemented	+	-	- (?)
3.	Breast cancer	+ (1)	-	-	-	-	-	Already implemented	+	.	- (?)
4.	Chronic Obstructive Pulmonary Disease	+ (1)	+	-	-	+	-	One-stop-shop is no solution for the existing bottleneck	+	+	- (?)
5.	Colon cancer	+ (4)	+	+	+	-	+	-	+	-	- (?)
6.	Colon cancer	+ (1)	+	+	-	-	+	-	+	-	- (?)
7.	Colon cancer	- (0)	+	+	+	-	+	-	+	+/-*	- (?)
8.	Head- and neck cancer	+ (7)	+	+	+	+	+	-	+	-	- (?)
9.	Hematuria	+ (2)	+	+	-	-	+	-	+	.	- (?)
10.	Lung cancer	+ (2)	+	-	+	-	+/-	Three-stop-shop	+	+	- (?)
11.	Oesophageal atresia (children)	- (0)	+	n.a.	-	-	-	n.a.	+	-	- (?)
12.	Open Chest Surgery	+ (6)	+	n.a.	+	+	-	n.a.	+	-	- (?)
13.	Small Orthopaedic interventions	+ (2)	+	+	+	+	+	-	+	+	- (?)
14.	Small Orthopaedic interventions	+ (3)	+	+	-	+	+	-	+	+	- (?)
15.	Benign Prostate Hypertrophy	+ (2)	+	+	-	-	+	-	+	-	+ (+)
16.	Colon cancer	+ (5)	+	+	+	-	+/-	Three-stop-shop	+	-	+ (+)
17.	Varicose veins	+ (5)	+	+	-	-	+	-	+	-	+ (+)

^1 ^Data source: survey among project staff members. ^2 ^Data source: survey among project leaders. ^3 ^Data source: interviews among project staff members. ^4 ^Data source: Sneller Beter database. + Yes, - No, . missing data, n.a. non applicable, because project only involves in-hospital care. * This project team used PDSA for testing and implementing a selection of the changes.

### Step three

After setting aims, the next step was to establish measures that would indicate whether a change led to an improvement. With one exception, all project teams made use of one or more of the outcome measures provided for the effect measurement. The provided intermediate measure was used by eleven project teams (Table 2). For three teams, this measure (number of visits to outpatient clinic) was not applicable because these projects involved only the redesign of in-hospital stay. For two project teams, the provided intermediate measure was not applicable because it was not related to the project aims: namely, the project did not strive to reduce the number of visits.

Eight project teams established additional outcome measures: for example, time between several diagnostic examinations within the diagnostic trajectory. Six project teams appointed intermediate and/or process measures to establish whether a process change was accomplished, for instance: Is the date of surgery planned directly after setting the diagnosis, yes or no? Five projects used no additional intermediate or process measure at all. Reasons for not using project-specific measures were that teams thought the provided measures gave enough insight to know whether a change is an improvement or because their project aims were not considered measurable (*e.g.*, qualitative aims such as a standardised discharge planning, or appointing one contact person for the patient during the whole care process).

### Step four

The main change idea, the one-stop-shop, presented in the collaborative meetings was applicable for 11 project teams (Table 2). Two of them did not succeed in combining the visits in one day due to organisational characteristics, the nature of the needed diagnostics, and/or the burden of the diagnostics to the patients. Six project teams thought the evidence was not applicable because they already combined all visits in the diagnostic trajectory into one; they did not redesign a diagnostic trajectory at the outpatients' clinic; or the long throughput time was not a result of many visits but of a long waiting list for one specific diagnostic examination. All project teams applied one or more of the other provided change concepts to redesign their care processes. Application of these change ideas required that project teams first investigated the causes of waiting times and delays in the redesigned process and then tailored the change ideas to their own setting. However, according to the project staff, tailoring change ideas proved more difficult in care processes in which more medical departments were involved, and accordingly more disagreement existed between the involved medical departments about the changes that had to be made.

### Steps five and six

During the interviews, project staff members were asked whether they had applied the PDSA cycle for change. Five confirmed that their project team used or was going to use the PDSA cycle. However, these five project teams did not split up every planned change in smaller changes as the change agent suggested. Further, staff members of these five project teams indicated that the PDSA cycle was not or would not be performed in a rapid cyclical mode because both the preparation for the test as well as the test of the change itself was time consuming. Because the patient groups were relatively small, a testing cycle took considerable time even when the number of patients per testing period was scaled down. The use of the PDSA cycle was also hampered by the fact that hospital information systems proved unable to generate data on the appointed measures when more hospital departments were involved. As a consequence, project teams had to gather data by hand, which was time consuming.

The teams that did not use or were not going to use PDSA for implementation (n = 10) chose to change the organisation of the care process radically by implementing their 'newly designed process' at once without first testing the individual changes. According to these project teams, testing change ideas within a short timeframe was not applicable to their situation because of the number of medical departments involved and/or the small number of patients involved in their redesign. Another reason for not testing in rapid cycles was the feeling that a test could fail due to non-optimal conditions when supporting processes were not optimised. For example, the team implementing changes in the care for open chest surgery patients considered it impossible to test a new operating room planning process. Changing the planning system for the operating room would necessitate adjusting all the supporting processes, including the working hours of the teams and how the rooms were prepared. Any testing before the altering of supporting processes would be massively disruptive.

### Step seven

Three project teams performed an effect measurement and reached collaborative goals (Table 2). The other project teams, including those that used the PDSA cycle, had not yet measured any interim results by December 2007 (one year after the start of the QIC). Therefore it is unknown whether they reached the collaborative goals.

From this description of the collaborative process we can identify several difficulties experienced by the project teams in applying the QIC method to process redesign. First, the adoption of change ideas and the accompanying measures provided by the external change agent, appeared not (directly) applicable for these collaborative project teams. Project teams had to tailor change ideas to their own context or could not use the provided change ideas at all.

Second, the adoption of the model for improvement by the project teams was hampered. Project teams were not capable of testing change ideas within a short time frame using PDSA cycles due to: the need for tailoring change ideas to their own context, and the complexity of aligning several interests of involved medical departments; the small volumes of the involved patient groups; and hospital information systems that proved unable to generate data on the appointed measures.

Third, project teams did not experience peer stimulus. All collaborative project teams intended to make improvements on an administrative subject, but in different parts of care processes (access times, throughput times of diagnostic trajectories, and/or length of stay) for different types of patient groups. As a consequence, project teams saw few similarities between their projects, rarely shared experiences, and demonstrated no competitive behaviour.

Further, a number of project teams perceived a lack of organisational support and external change agent support. However, the project teams that succeeded in implementing changes (projects 15, 16, and 17) shared the opinion that preconditions for successful use of the QIC method--*i.e.*, 'team organisation', 'organisational support', and 'external change agent support'--were in general sufficiently present. Only organisational support lacked in one of the three project teams (project 15).

## Discussion

From the results it seems that in the evaluated collaborative the QIC method was not used. Apparently, it did not contribute to empower project teams to implement their process redesign in a short timeframe. As a consequence, this study could not show whether the QIC method can effectively contribute to process redesign, if used. The description of the collaborative process provides us with valuable information about the difficulties experienced by the project teams in applying the QIC method to process redesign. In this section, we will discuss explanations for these difficulties, which concentrate on a lack of fit between the QIC method and process redesign, a non-optimal application of the QIC method, and non-optimal conditions for using the QIC method.

### Non-optimal fit between the QIC method and process redesign

First, a lot of the project teams needed customised solutions for their process redesign, while the QIC method aims to spread standardised evidence-based practices or change ideas to serve many teams at the same time with a limited number of external change agents. According to the QIC method collaborative project teams should benefit of the exchange of the standardised change ideas in such a way that they can eliminate much of the investigative work on problem analysis and change ideas in comparison with traditional quality project teams [3]. For example, in a QIC for pressure ulcers, an external change agent can provide concrete best practices from pressure ulcer guidelines to perfect the elements of care, such as 'minimise skin pressure through the use of a positioning schedule for clients with an identified risk for pressure ulcer development'. This best practice can then be tested and, if it works, be implemented directly in every setting. Process redesign, however, calls for customised solutions because project teams need to handle context-specific causes of waiting times and delays in care processes determined by the existing interaction patterns between departments in their hospital. Project teams can therefore not test the standard change ideas provided by the change agent within a short time frame but have to investigate the causes of waiting times and delays and to tailor change ideas to their own setting. As a consequence, the collaborative cannot eliminate the investigative work on problem analysis and profit from standard change ideas provided by the external change agent as the QIC method prescribes.

Second, the model for improvement, and especially the PDSA cycle, seemed inappropriate to test intended changes within a short timeframe. The QIC method assumes that testing one big change idea lowers the resistance to a change because clinicians are more likely to be reassured that the change is effective [8,17]. This assumption ignores the fact that testing changes that affect several departments may lead to more consultation before testing a change and thus to an increased possibility of resistance to a change. This happened in the hospitals involved as result of their functional structure, in which every department has its own responsibilities and tries to optimise its own functioning. These functional boundaries hampered, for example, the adjustment of the department schedules needed to realise a 'one-stop-shop'. After all, more relationships are affected, and more different interests play a role. As a result, project teams could only start testing after a buy-in or political solution. In this study, the complexity of aligning department schedules and interests became more apparent when the number of departments involved in a care process increased. The project teams might have improved the collaboration across boundaries if they had included in their team a medical specialist from all medical department(s) involved. However, the need for buy-in solutions before testing a change could also be due to the fact that the external change agent advised splitting up every planned change into smaller changes. Although smaller changes can reduce the risk of failure, it also lowers the expectations of the benefits of a change. Unclear or smaller benefits do not stimulate medical departments to invest in making changes.

Difficulties in using the PDSA cycle meant that most teams decided to implement changes without testing them. Subsequently, teams did not get feedback on the work they were doing and did not experience a momentum of change [18]. It is known from previous studies that consistent ongoing measurement is required to tell whether changes being made are leading to an improvement, and to provide basis for continued action [19,20]. Because of this lack of feedback, teams were not stimulated to adapt another change idea for improvement, which in turn slowed down the implementation of changes.

Although the difficulties with the use of the PDSA cycle are (almost) inevitable in process redesign projects in functionally organised hospitals, the use of the PDSA could be improved by taking care of some preconditions. First, hospital information systems should be able to generate data on the appointed measures. Second, the number of patients involved in the care process that need to be redesigned has to be big enough to test a change idea within a number of days.

### Non-optimal application of the QIC method

Next to the non-optimal fit between the QIC method and process redesign, difficulties can also be due to the selection process of the collaborative project teams. The external change agent included project teams in the collaborative that worked on different parts of care processes (access times, throughput times of diagnostic trajectories, and/or length of stay) for different types of patient groups, while the QIC method aims to implement evidence-based practice through sharing knowledge with others in a similar setting [4]. Probably, the external change agent could have provided peer stimulus if it had selected project teams that worked on comparable process redesign projects with comparable goals. Nevertheless, lack of peer stimulus can also occur between comparable redesign projects because of the existence of context-specific causes of delays and waiting times.

### Non-optimal conditions for using the QIC method

Next to hospital information systems to generate data on outcome, intermediate and process measures, complex process redesign projects need support to change interaction patterns between involved departments. A number of project teams perceived a lack of organisational and external change agent support, despite the facts that all project teams received external change agent support and the participating hospitals were enrolled in the 'Sneller Beter' programme by a selection procedure that assessed the organisational support. Unfortunately, we could not identify factors that contributed to this perceived lack of organisational and external change agent support.

### Limitations

This study aimed to assess the applicability of the QIC method for process redesign. Although we think the findings of this study provide useful information for future collaboratives, the results need to be interpreted with caution. The findings of this evaluation could be influenced negatively by the selection process of both the collaborative project teams and the care processes to be redesigned. For instance, not all teams participated in the collaborative on a voluntary basis. Unfortunately, we could not determine with certainty to which project teams this applied and how this influenced the collaborative process.

Another limitation is that the gathered data are not complete. However, observations during meetings and training sessions of the process redesign collaborative showed us that the missing data of project leaders and project staff members are not related to poor performing project teams and/or organizational support. The poor availability of effect measurements on collaborative goals can be contributed to the fact that it is not feasible for many project teams to redesign, implement, and perform an effect measurement within a year, and to the non-optimal fit between the principles of the used QIC method and process redesign.

## Conclusion

This study showed that the need for tailoring standard change ideas to the context of collaborative project teams, and the complexity of aligning several interests of involved medical departments, hampered the use of the QIC method for process redesign. We cannot determine whether the QIC method is appropriate for process redesign. As result of the selection process for participation of project teams by the external change agent peer stimulus was non-optimal. Further project teams felt that preconditions for successful use of the QIC method were lacking. Therefore, additional research into the applicability of the QIC method for process redesign is needed.

## Competing interests

The authors declare that they have no competing interests.

## Authors' contributions

LV was responsible for designing the study, conducting the multiple case study, analyzing and interpreting the data, and drafting the manuscript. MD participated in the design of the study, assisted in interpreting the results, and drafting the manuscript. CW and GM participated in the design of the study, assisted in interpreting the results, the critical revision of the manuscript, and its supervision. All authors have read and approved the final manuscript.
